# Who Will Help to Strive Against the “Infodemic”? Reciprocity Norms Enforce the Information Sharing Accuracy of the Individuals

**DOI:** 10.3389/fpsyg.2022.919321

**Published:** 2022-06-30

**Authors:** Kehan Li, Weiwei Xiao

**Affiliations:** ^1^School of Economics, Shandong University of Finance and Economics, Jinan, China; ^2^The Center for Economic Research, Shandong University, Jinan, China

**Keywords:** information accuracy, information sharing, reciprocity norms, feedback, experiment

## Abstract

In recent years, misinformation sharing has become the focus of public debate and academic research. We aim to explore whether individuals prefer to share accurate information or not, and discover what factors increase people’s preferences for sharing accurate information. Combining behavioral economics experiments and psychology experiments, we construct “an information search—information sharing—information feedback experiment” to examine individuals’ behavior of sharing accurate information and its influencing factors. A total of 210 students are recruited for the laboratory experiment. Our results show that when individuals can control the accuracy of the information they obtain through their efforts, they are more willing to share accurate information with others. We also find that positive feedback from information receivers can promote the accuracy of information shared by individuals, and this effect works through reciprocity norms. Individuals with higher reciprocity are more willing to share accurate information, especially in the treatment with the role of reciprocity norms enhanced by feedback. These findings indicate that individuals who are willing to obtain accurate information prefer to share information, and information feedback can enhance this preference through reciprocity norms. This study captures individuals’ behavior and preference characteristics with regard to the accuracy of the information they share in the era of highly developed network interaction.

## Introduction

The rapid innovation of Internet technology and the low threshold and ease of use of network interaction technology have greatly changed the role of individuals in information exchange. Specifically, the role of individuals has been rapidly changing from the end point of acquiring and receiving information or the starting point of sharing information to the node on the information network. Social Media offer users the opportunity to be both receivers and publishers. In the age of greatly abundant information, the spread of inaccurate information, misinformation and fake news has always been seen as a threat to science and society (Cuan-Baltazar et al., 2020; Kozyreva et al., 2020; Lewandowsky and van der Linden, 2021). Inaccurate or misinformation will lead to incorrect beliefs, which in turn induce a series of social problems such as harmful consequences on topics ranging from COVID-19 to the 2020 election of the U.S. (Pennycook et al., 2020; Yi et al., 2021). For example, misinformation about COVID-19 possibly causes serious harm such as raising anxiety levels (Dobson-Lohman and Potcovaru, 2020), and it can it can easily spread to other people because fear and anxiety are contagious (Lzroiu et al., 2020). Spreading false cures or unfounded preventive measures (Swire-Thompson and Lazer, 2019; Mian and Khan, 2020; Saling et al., 2021) reduces people’s willingness to comply with scientific health measures such as vaccinations or wearing masks (Rommer et al., 2020; Loomba et al., 2021). Dr. Tedros Adhanom Ghebreyesus, WHO Director-General even pointed out that “We’re not just fighting an epidemic; we are fighting an infodemic” (Tedros, 2020).

In recent years, the misinformation sharing has become a major focus of public debate and academic research (Lazer et al., 2018; Pennycook et al., 2021). Scholars have explored the psychological motivations for people to spread and share misinformation or fake news (Grinberg et al., 2019; Bryanov and Vziatysheva, 2021; Pennycook and Rand, 2021). A popular claim is that fake news sharing has its roots in politics bias (Lazer et al., 2018; Allcott et al., 2019; Lewandowsky and van der Linden, 2021; Weismueller et al., 2022). For example, Michael and Breaux (2021) find that political affiliation influence people’s descriptions and their beliefs about which news sources are “fake.” Osmundsen et al. (2021) argue that much fake news in Western societies stems from a need to denigrate political opponents. Moreover, people may share interesting, thrilling but inaccurate information purely for social, entertainment, or attention-seeking needs (Altay et al., 2022). In fact, misinformation tends to be more related to human prejudice such as counter intuition, threat, hatred, sex, etc., than accurate information, aiming to support social interactions such as gossip, cheating, formation of alliances, etc. (Acerbi, 2019). Some studies have found that there are individuals in society who simply desire chaos, they “want to see the world burn down” (Arceneaux et al., 2021), and thus are more inclined to share uncertain information such as “conspiracy theories” (Douglas et al., 2017; Bratu, 2020; Sheares et al., 2020; Prooijen et al., 2022).

Recently, some studies retrospectively explain the reasons for sharing misinformation from the perspective of information accuracy (Chambers, 2021; Pennycook et al., 2021; Pennycook and Rand, 2021; Altay et al., 2022). They find that political bias, social needs, or chaos desire have an effect on misinformation sharing, and ignoring the information accuracy may play a key role in sharing misinformation (Pennycook and Rand, 2021). Moreover, reminding or asking people about the accuracy of information can inhibit the sharing of misinformation (Pennycook et al., 2021; Roozenbeek et al., 2021). The premise of this conclusion is that people care about the information accuracy when sharing information. Based on this, we first explore whether people prefer to share more accurate information if they can control the accuracy of their own information in situations where information accuracy is directly related to personal benefits. In response to this question, an information search—information sharing laboratory experiment is performed, in which participants are required to search information at a cost, and the accuracy of the information they search for directly affects the probability of obtaining high rewards, and then they need to decide whether to share the information with the rest of the group at a cost.

We are more interested in the factors that influence people’s behavior of sharing accurate information. In terms of information sharing, reciprocity norms are considered to be a key factor in encouraging people to share information voluntarily and promoting information dissemination (Hsu and Lin, 2008; Schumann et al., 2014; Pai and Tsai, 2016). As a kind of universal social norms, reciprocity norms enables both sides to enhance mutual assistance (Cook et al., 2013). In other words, motivated by reciprocity norms people are able to voluntarily help others and share information (Kim et al., 2021), even without direct reciprocity (Lee and Suzuki, 2020). Based on the promotion effect of reciprocity norms on information sharing, we further explore whether reciprocity norms promote the sharing of accurate information, that is to say, whether individuals with higher reciprocity are more willing to share accurate information they obtain. In response to this question, the participants’ reciprocity preferences are measured, and a feedback procedure is added to the previous information search—information sharing experiment. Hence, an information search—information sharing—information feedback experiment is constructed. That is, after sharing information, participants can get positive feedback (such as clicking likes) from its information receivers. The feedback is considered to enhance the promotion effect of reciprocity norms on information sharing and promote individuals’ information sharing behavior in the future (Lee and Suzuki, 2020). Based on the two experiment treatments (treatment with feedback and treatment without feedback), we attempt to explore the following questions in the context of people collecting information themselves and sharing it: (a) do people prefer to share accurate information? (b) Does the reciprocity norms promote the sharing of accurate information?

## Theoretical Background and Hypotheses Development

### Information Sharing, Misinformation and Information Accuracy

In the current information age, the reason why Twitter, Tencent, Tik Tok, etc., have become half of the Internet world is that they have captured people’s fundamental preferences to some extent for communication desire, i.e., information sharing behavior. Hence, attentions have been paid for the impacts of information sharing on the performance of teams, organizations, businesses, etc. (Cummings, 2004; Hsu, 2008; Wang and Wang, 2012). Studies suggests that it can promote innovation, enhance organizational competitive advantages, and even increase social equality (Kang and Lee, 2017; Qureshi et al., 2017). The maturity of Internet information technology and long-term information and knowledge management practices make it more and more unquestionable that information and knowledge sharing can promote performance and social welfare (Hung et al., 2011; Park and Gabbard, 2018).

However, in recent years, the possible negative effects of information sharing have attracted attentions, especially the misinformation sharing (Pennycook and Rand, 2021). Scholars even argue that we are living in a “post-truth” era (Lewandowsky et al., 2017; Ball, 2018). Economic inequality, increased political polarization, diminished trust in science, and an increasingly fragmented media landscape are considered as the presentations of this era (Pennycook et al., 2021). Misinformation and fake news spread faster than accurate information because of its cognitive salience and attractiveness (Acerbi, 2019). Misinformation will distort public perceptions, thus reducing people’s trust in universal sources of information, anchoring biased beliefs about unfamiliar people or things, etc. (Murphy et al., 2019; Jost et al., 2020; Ognyanova et al., 2020). Studies find that despite the cognitive appeal of uncertain information or fake news, both ordinary people and those who are wary of the threat posed by inaccurate information will overwhelmingly value accuracy (Chambers, 2021; Altay et al., 2022). In this case, more attention has been paid to the accuracy of information.

In general, accuracy is one of the determinants of the quality of information sharing (Nicolaou et al., 2013). Existing literature have shown that people do not prefer to share less accurate information (Pennycook et al., 2020). There are other reasons for sharing and disseminating inaccurate information. One alternative explanation is that people’s ability to judge the accuracy of information is insufficient. Serra-Garcia and Gneezy (2021) find that people have difficulty judging uncertainty in information, but they are overconfident in their ability to judge the accuracy of information, so that when people are motivated to share accurate information, this information may instead be inaccurate. Yet more researches consider that most people are able to correctly assess the accuracy of information (Bago et al., 2020; Pennycook et al., 2020, 2021; Pennycook and Rand, 2021). The sharing of inaccurate information is not due to people’s inability to assess the information accuracy, but the inability to allow accuracy judgments to determine the information sharing. That is, although most people do not want to share uncertain information, their attention is diverted from accuracy by some other significant motivations when people choose to share information (Altay et al., 2022). Based on this, guiding people to think about the accuracy of the information they see can help reduce misinformation sharing (Pennycook et al., 2021; Roozenbeek et al., 2021). The above analyses rely on a premise that people are indeed prefer to share accurate information than inaccurate information (Pennycook et al., 2021). In our experiments, participants need to choose one item from a set of N items as the final payment basis. However, the information of each item is hidden behind M boxes with a question mark. The participants costly select the boxes they want to turn over to acquire item information. Participants are more likely to select the row with the greatest payoffs if more question mark boxes are opened. Thus, participants can determine the information accuracy themselves by information search. We argue that in our experiment, participants are more willing to share more accurate information when they are able to control the accuracy of the information. Accordingly, the following hypothesis is proposed:

*H1*: People prefer to share accurate information they obtain.

### Reciprocity Norms and Accurate Information Sharing

Reciprocity norms are generally considered to be the decisive driving force for information sharing (Haeussler, 2011; Schumann et al., 2014; Pai and Tsai, 2016). Lin (2007) even argues that without reciprocity norms, information sharing will not develop. Reciprocity norms emphasize a person’s obligation to reciprocate favors given to him/her by others (Haeussler, 2011). Studies have found that people tend to share information to give back the help of others (Faraj and Johnson, 2011; Wu and Korfiatis, 2013). In the social context established by reciprocity norms, social-psychological factors of anticipated reciprocity are one of the driving forces for information sharing (Zaheer and Trkman, 2017). Individuals often share information and knowledge in the expectation of returns, such as material benefits, information exchange, self-satisfaction, etc. (Kim et al., 2021). This means that an individual is willing to share information as long as he/she expects that others will provide information or other feedback in exchange (Zaheer and Trkman, 2017). For instance, Lee and Suzuki (2020) find that other members’ positive expressions (such as *likes*) on previously shared information will promote future information sharing, which is achieved through reciprocity norms; Pai and Tsai (2016) consider that the role of reciprocity norms in promoting information sharing requires effective development of social-, hedonic-, and utilitarian-focused drivers.

Reciprocity norm greatly promotes people’s willingness and behavior to share information (Zaheer and Trkman, 2017). In social networks, the exchange of resources and support occurs in interactions, the behavior of information sharing may affect the individuals participate in the interactions or others who observe the exchange (Starr et al., 2020), which forms the reputation of the information sharers. Generally speaking, reputation is often considered as the external driving factor of individual information sharing behavior. The drive is considered to derive from the reward of the reputation formed by information sharing (Chang and Chuang, 2011; Park et al., 2014). These rewards are obtained through reciprocity norms (Lee and Suzuki, 2020), and individuals with high reputations are more attractive or more likely to get help (Gintis et al., 2001). The relationship between reputation and reciprocity is often thought to be bidirectional. A higher reputation leads to a higher reciprocity, and a higher reciprocity leads to a higher reputation (Starr et al., 2020).

The relationship between reciprocity and information accuracy is bridged by reputation. Altay et al. (2020) show that despite the attractiveness of inaccurate information, most people tend to avoid sharing inaccurate information because they want to maintain a good cognitive reputation, thereby to obtain social rewards that reputation may bring. Further, Ecker et al. (2022) argue that people are concerned with accuracy to a certain extent. When people have to share inaccurate information, they need to be paid because they worry that sharing inaccurate information may incur reputational costs (Waruwu et al., 2021). From this perspective, individuals who perceive reciprocity norms more strongly, or are more influenced by social-psychological factors of anticipated reciprocity, are more likely to value the reciprocal rewards of a good reputation, that is, they place more value on the accuracy of information when sharing information. Based on the above analysis, the following hypotheses are proposed:

*H2*: Individuals with stronger perceptions of reciprocity norms prefer to share information.

*H3*: Individuals with stronger perceptions of reciprocity norms share information with a higher accuracy.

## Materials and Methods

### Overview of the Study

To explore people’s willingness to share accurate information when they can control the accuracy of the information they obtain, we design an information search—information sharing (treatment 1) laboratory experiment. To further analyze the possible promotion effect of reciprocity norms on accurate information sharing behavior, the procedure of positive feedback from information receivers is added to perform an experiment of information search—information sharing—information feedback (treatment 2).

**Figure 1:**
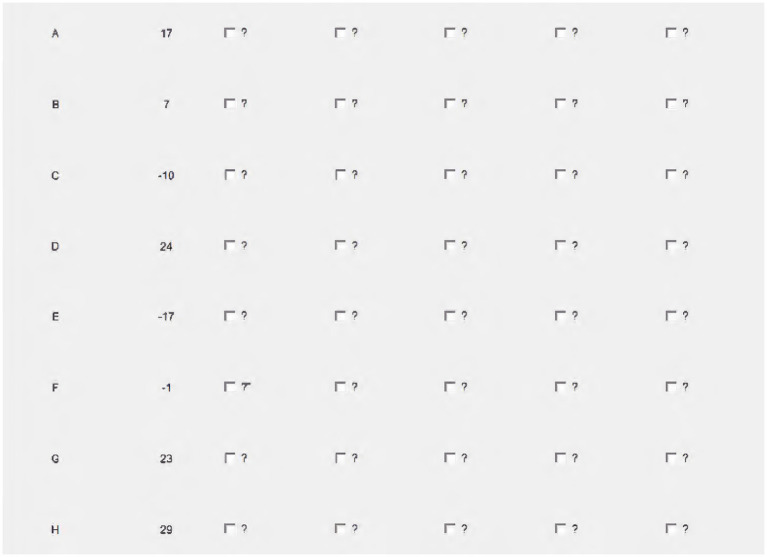
Selection game of *N* items.

The information search part of the experiment is adapted from the information search experiment by James et al. (2006). In the experiment, participants select one item from a set of N items, each of which has *M* pieces of information hidden behind M boxes with a question mark on the computer screen. That is, participants face the question mark boxes distributed in a matrix of *N* rows × *M* columns (see Figure 1). The participants need to select the boxes they want to turn over from the *N* × *M* boxes. After confirming, all the chosen boxes will be turned over to show the hidden information, and the boxes that have not been turned over remain with a question mark. After opening boxes for weighing, the participants select a row from the *N* rows as the final payment basis, and the numbers in the *M* boxes in this row will be summed up as the final payment regardless of whether they are selected or not. In our experiment, the participants need to pay a cost to each box they turned over, which is different from the experiment of James et al. (2006). Thus, on the one hand, participants are more likely to select the row with the greatest payoffs if more question mark boxes are opened (James et al., 2006). On the other hand, participants need to make tradeoffs between the benefit of information accuracy and the cost of information search.

After the information search stage adopted from the experiment of James et al. (2006), we add an information sharing stage in which participants decide whether to share the information to others they costly obtained in the information search stage.

In our experiment with *N* = 8 and *M* = 6, participants who need to search for information in the experiment will see a matrix with 8 (rows) × 6 (columns), as shown in Figure 1. The first column has given the 8 numbers and the remaining numbers are covered by question marks. Participants need to decide how many boxes to open, and which ones to open. Each of the 6 columns of numbers is randomly drawn from a normal distribution. The first column gives the distribution of the numbers: *N*(10, 20.4) and the numbers drawn from this distribution can make a 95% confidence interval of −30 ~ 50. From left to right, the normal distribution of the numbers drawn from each column is expected to be unchanged, and the variance decreases by 1/6 in turn. That is to say, the numbers in the second column obey the normal distribution of, *N*(10, 18.62) the third column obeys the distribution of, N(10, 16.66) and so on, and the last column obeys the normal distribution of *N*(10, 8.33) Based on the previous analysis, the number of individuals’ information searches can be used as a proxy for the accuracy of the information they obtain.

### Participants

A total of 210 students are recruited for the experiment, all of whom are postgraduates or senior undergraduates. Among them, 47.14% are female, 45.71% are postgraduates, and 45.71% are majored in MBA and management. The experiment platform is Z-tree software. Each round of the experiment takes about 70 min, and the average payment per person is 32.18 yuan. A total of 7 experiment sessions are conducted, of which 3 are treatment 1 used to examine the relationship between information search and information sharing behavior of sharers when the information receiver does not give feedback. The number of participants is 27, 45 and 36, respectively. The other 4 are treatment 2, which are used to examine the relationship between information search and information sharing behavior of sharers when information receivers give feedback. The number of participants is 21, 36, 27 and 18, respectively.

### Materials and Procedure

#### Treatment 1: Experiment Without Feedback

Specifically, at the beginning of the experiment, all participants are informed that there are two types of participants, A and B. The participant type is randomly determined and will not change throughout the experiment. One A and two Bs form a group, a total of three participants. The participants will not know the other two participants in their group. The three participants who make up the group in each round of the experiment will be randomly assigned to simulate an interaction situation. Participants are not told how many rounds the experiment will be conducted to rule out deadline effects, and each round is divided into 2 phases.

Phase 1 is the information search. Type A participant obtains an initial fund of 80 G$ (Game Dollar, G$ for short), and can choose to open the boxes with question marks. The numbers contained in the boxes will be displayed on the next screen, and the boxes not be opened will still be displayed as “?” on the next screen. Opening a question mark box requires a cost of 2 G$. That is to say, if participant A wants to open all 40 boxes, then he/she needs to pay a cost of 80G$. Type B participants have no power to open the box and thus do not need to make decisions during Phase 1.

After phase 1, A needs to decide whether to tell the information he/she has searched to B in the group. If choosing to share with B, A needs to pay a cost of 5 G$. If not, he/she does not need to pay. After that, A needs to choose a row from the 8 rows, and the 6 numbers in the chosen row are added up as part of A’s final earnings.

The final earnings function of A in this round is as follows:

A’s earnings = 80 G$-2 G$ × the number of boxes opened+ the sum (G$) of the 6 numbers in the chosen row

- $\{\begin{array}{c} 5\;\mathrm{G\$},\mathrm{If}\;A\;\mathrm{decides to share the information with}\;B\\ 0,\mathrm{If not}. \end{array}$

Next, the experiment enters phase 2. B is given an initial funding of 30 G$, and will know whether A in the group shares the information he/she has searched. If A chooses to share the information, then B will see the information searched by A. If not, then B will see the initial interface with 40 question marks.

B needs to select a row from the 8 rows. The 6 numbers in the selected row are added up as part of B’s final earnings. The earnings function of B is as follows:

B’s earnings =30 G$ + the sum (G$) of the 6 numbers in the selected row

#### Treatment 2: Experiment With Feedback

The first two phases of treatment 2 are the same as treatment 1, but there is one more phase. After phase 2, B can spend 3 G$ to give positive feedback to A, or return nothing at no cost. The final earnings function of B is as follows:

B’s earnings =30 G$ + the sum of the 6 numbers in the chosen row

- $\{\begin{array}{c} 3\;\mathrm{G\$},\mathrm{If}\;B\;\mathrm{decides to give feedback}\;\\ 0,\mathrm{If not} \end{array}$

Before the experiment, a pre-test is conducted, in which 20 volunteers are asked to score 20 positive feedback sentences. 10 sentences with the highest positive scores make up the feedback sentence library of B.

After all rounds of the experiment, participants are asked to fill out a reciprocity norms questionnaire, as well as a demographic questionnaire.

### Measurements

#### Dependent Variable

##### Information Sharing Behavior

Whether participants share the information they acquired during the experiment. It is coded as “0” if participants do not share their acquired information, and otherwise “1.”

#### Independent Variables

##### Information Accuracy

the accuracy of information obtained by participants in the information search stage. It is measured by the number of information searches.

##### Norm of Reciprocity

It is measured using the Norm of Reciprocity Questionnaire adapted from Chen et al. (2018) and Han and Wibral (2020), including three questions “If someone else shared information with me before, I am ready to reciprocate him/her,” “When I receive information from the group, I feel right to share and help others,” and “If I’ve been treated well by others in the past, I feel I have a responsibility to help others at a personal cost.” These questions are measured using a 7-point Likert scale. A higher score indicates that the individual has stronger reciprocity norms. In the Questionnaire, Cronbach’s *α* = 86.9%, the standard error is 0.035.

##### Feedback Treatment

Whether the participant participates in the experimental treatment with positive feedback. It is coded as “0” if the participant is in the treatment 1 without positive feedback, and otherwise “1” if the participant participates in the treatment 2 with positive feedback.

#### Control Variables

We control for the personal information given by the participants during the experiment. See notes of Table 1 for the specific coding rules of the dummy variables of participants’ demographic characteristics. Table 1 reports the descriptive statistics of participants’ demographic characteristics, including gender, grade, major, and political affiliation, etc. As not all variables are normally distributed, we report the medians and interquartile ranges of the variables.

**Table 1:** Descriptive statistics.

Variable	Full sample		Treatment 1		Treatment 2	
	Median	IQR	Median	IQR	Median	IQR
Gender	0	1	1	1	0	1
Grade	1	1	2	1	1	1
Major	0	1	0	1	0	1
Work experience	0	0	0	0	0	0
Academic performance ranking	2	1	2	1	2	1
Political affiliation	2	2	2	2	2	2
Average monthly household income	2	2	2	2	2	1

*The dummy variables in the table are coded as follows: (1) Gender: female = 1 and male = 0; (2) Grade: undergraduate = 1 and master = 2; (3) Major: economics and management majors = 1, and non-economics and management majors = 0; Work experience: no work = 0, 1 year or less = 1, and 1 year to 3 years = 2; Academic performance ranking: ranking in the top 20% = 3, ranking between the top 20 and 50% = 2, and ranking in the bottom 50% = 1; Political affiliation: nonparty personage = 1, a member of the league = 2, a member of a democratic party = 3, and a member of the Communist Party of China = 4; Average monthly household income: less than 2,000 yuan = 1, 2,000 yuan to 5,000 yuan = 2, 5,000 yuan to 10,000 yuan = 3, and more than 10,000 yuan = 4.*

### Analysis of Experimental Results

#### Information Accuracy Based on Information Search Behavior

Firstly, individuals’ information search behavior is analyzed so as to discuss the relationship between the opened information and the individuals’ subsequent search behavior. The numbers in the first column are sorted with the largest number ranked as “1,” and the smallest number ranked as “8.” The ranking is taken as the independent variable and the number of question marks in the corresponding row after the participant number as the dependent variable to perform regression analysis. Meanwhile, individuals’ identity labels and the experiment sessions are controlled. Table 2 reports the Logit regression results. In this study, the number of individuals’ information searches is used to represent the accuracy of the information possessed by the individuals. A greater number of question marks individuals turn over indicate that it is more likely to choose the optimal payment row. In order to verify the feasibility of this method, the regression is carried out with whether the individual make the optimal choice as the dependent variable, and the number of question marks turned over by individuals as the independent variable. The results are shown in Table 2. The experimental results imply that the more the boxes turned over by individuals, the more likely they are to select the optimal payment row (coef. = 0.0747, *p* < 0.01). The width of the 95% confidence intervals [0.0513, 0.0981] is small which confirms the effect of association. Thus, it is reasonable to use the number of information searches as a proxy for information accuracy.

**Table 2:** Regression analysis of information search behavior.

Independent variable	Optimal choice or not
	Full sample
Information accuracy	0.07466^***^
	(0.01194)
Gender	0.3165^***^
	(0.06577)
Grade	0.1945^***^
	(0.06933)
Major	0.2408^***^
	(0.06654)
Work experience	−0.2342^***^
	(0.06780)
Academic performance ranking	−0.1490^***^
	(0.04361)
Political affiliation	0.02398
	(0.02972)
Average monthly household income	0.1742^***^
	(0.03328)
Constant value	0.3165^***^
	(0.06577)
Sample size	700
*R*^2^ (Pseudo *R*^2^)	0.076

(1) Treatment is a dummy variable representing experiment treatment. 0 means treatment 1, 1 means treatment 2. (2) ^***^p < 0.01.

#### Information Accuracy and Information Sharing Behavior

The experiment involves important decisions in the two phases, namely, information accuracy determination, and information sharing decisions. The regression is first used to examine the influence of individual’s demographic characteristics such as gender, grade, major, work experience, academic performance ranking, political affiliation, and average monthly household income on individual information sharing behavior. After controlling for these demographic characteristics, regression is performed on the relationship between information accuracy and participants’ information sharing behavior. First, the information accuracy is taken as the independent variable, and the information sharing behavior as the dependent variable for regression. Table 3 reports the Logit regression results. Because the experiment is divided into treatment 1 without feedback from the information receivers and treatment 2 with feedback, sub-sample regression is also performed on the treatments 1 and 2, respectively, in addition to the overall regression.

**Table 3:** Information accuracy and information sharing behavior.

Dependent variable: information sharing behavior						
Independent variable	Overall regression			Treatment 1		Treatment 2	
	Regression 1	Regression 2	Regression 3	Regression 4	Regression 5	Regression 6	Regression 7
Information accuracy		0.04836^***^	0.05109^***^	0.03222^***^	0.04245^***^	0.08143^***^	0.07946^***^
		(0.003666)	(0.003761)	(0.004434)	(0.004990)	(0.005486)	(0.005699)
Gender	0.5424^***^		0.6384^***^		1.6158^***^		0.1576^*^
	(0.06529)		(0.06693)		(0.1452)		(0.09453)
Grade	0.4949^***^		0.6350^***^		1.3392^***^		−0.005639
	(0.06621)		(0.06751)		(0.1215)		(0.1056)
Major	−0.7929^***^		−0.6464^***^		−0.5737^***^		−0.2681^***^
	(0.06338)		(0.06481)		(0.1226)		(0.09264)
Work experience	−0.2492^***^		−0.1737^**^		0.1484		−0.5861^***^
	(0.07530)		(0.07255)		(0.1721)		(0.09774)
Academic performance ranking	0.03206		0.1008^**^		−0.7284^***^		0.4393^***^
	(0.04557)		(0.04624)		(0.07746)		(0.06636)
Political affiliation	−0.02063		−0.1112^***^		−0.08730		−0.008860
	(0.03076)		(0.03136)		(0.05577)		(0.04886)
Average monthly household income	−0.03908		−0.05188		0.03943		0.07658^*^
	(0.03337)		(0.03390)		(0.05740)		(0.04530)
Treatment	0.4562^***^	0.3163^***^	0.5022^***^				
	(0.06035)	(0.05495)	(0.06019)				
Constant value	−0.4540^***^	−0.4310^***^	−1.2630^***^	−0.2588^***^	−1.2981^***^	−0.4415^***^	−1.3945^***^
	(0.1646)	(0.05078)	(0.1743)	(0.05871)	(0.2978)	(0.06818)	(0.2480)
Sample size	700	700	700	360	360	340	340
Pseudo *R*^2^	0.0716	0.0346	0.1026	0.0173	0.1788	0.0572	0.0923

(1) Information sharing behavior is determined by whether the participant is willing to share the searched information to the information receivers in the same group in the phase 2 (unwilling to share = 0; willing to share = 1). Treatment is a dummy variable of experiment treatment (treatment 1 = 0; treatment 2 = 1). (2) ^*^p < 0.1, ^**^p < 0.05, and ^***^p < 0.01.

In Table 3, regression 1 show that participants’ work experience, academic performance ranking, political affiliation, and average monthly household income have low significant effect on their information sharing preferences. Gender is significantly correlated to sharing preferences at the 0.01 level with 95% confidence interval of [0.1802, 0.9046]. The width of the 95% confidence intervals is too large to confirm the effect size of the regression. That is, the difference between females’ information sharing behaviors and males’ behaviors show weak effect. Grade is also positively correlated to participants’ sharing behaviors with 95% confidence interval of [0.1276, 0.8622] which also indicate a weak association. In addition, major background is significantly negatively correlated to the willingness to share information with a small width of 95% confidence interval which is [−1.1444, −0.4413]. Individuals majored in economics and management is more reluctant to share information. From regressions 2 and 3, it can be seen that, overall, the individual information sharing behavior is significantly positively correlated to the information accuracy at the 0.01 level (95% CI = [0.03023, 0.07195]). That is, people with higher information accuracy are more willing to share information. From regressions 4 to 7, it can be seen that regardless of whether the information receivers give feedback, people’s willingness to share accurate information do not change, and it is significant at the 0.01 level. The widths of the 95% confidence intervals of these regressions (95% CI = regression 5, [0.01475, 0.07014]; regression 7, [0.04783, 0.1111]) indicate high effects of the regressions. Further, compared with the situation in which the information receiver does not give feedback, the correlation coefficient between the information accuracy and the sharing behavior is larger when receiver gives feedback. That is to say, the sharing behavior of individuals may be enhanced by the accuracy of the information they possess.

#### The Influence of Feedback on Information Sharing Preferences

In treatment 1, the information sharers will not get feedback from the information receivers, while in treatment 2, the participants will get positive feedback expressing gratitude or appreciation from the information receivers. Comparing the sharing behaviors of participants in treatments 1 and 2, we can find the impact of positive information feedback on individual sharing preferences. Table 4 gives the parametric test results of individual sharing behaviors in the two experimental treatments. As the sample of individual sharing preferences passes the normality test (Treatment 1: *z* = −7.466, *p* > 0.05; Treatment 2: *z* = −3.576, p > 0.05) and the homogeneity test of variance (Chi-square = 0.1267, *p* > 0.05), we use T-test to perform the parametric test.

**Table 4:** Comparison of information sharing behavior in treatments 1 and 2.

Variable	Experimental treatment	Observations	Mean	Sub-sample *t*-test		Rank sum test	
				*t*	sig	*z*	sig
Information sharing behavior	Treatment 1: without feedback	360	0.5250 (0.02635)	−2.0015	0.0457		
	Treatment 2: with feedback	340	0.6000 (0.02661)				
Information accuracy	Treatment 1: without feedback	360	11.3306 (0.5265)			−0.5060	0.6126
	Treatment 2: with feedback	340	11.2176 (0.4564)				

It can be seen from Table 4 that when the information receivers give feedbacks expressing gratitude or appreciation to the information sharing participants, the sharing behaviors of the participants will be significantly improved (0.5250 v.s. 0.6000, *t* = −2.0015, *p* = 0.0457. diff 95% CI = [−0.1486, −0.0014]). In order to further verify this result, the dummy variable of whether to give information feedback is taken as the main independent variable. If the information receivers give feedback to the information sharer, the dummy variable equals 1; if not, it equals 0. Then, the information sharing preference is taken as the dependent variable for regression. Table 5 reports the regression results. Regressions 1 and 2 show that the treatment with feedback significantly positively correlated with information sharing preferences (coef. = 0.4575, *p* < 0.01, 95% CI = [0.1224, 0.7925]), indicating that individuals’ information sharing behaviors are enhanced when the information receivers give positive feedback.

**Table 5:** Relationships between feedback and information sharing behavior and information accuracy.

Dependent variable: information sharing behavior	
Independent variable	Regression 1	Regression 2
Feedback treatment	0.3054^**^	0.4575^***^
	(0.1531)	(0.1709)
Gender		0.5439^***^
		(0.1844)
Grade		0.4963^***^
		(0.1884)
Major		−0.7952^***^
		(0.1802)
Work experience		−0.2499
		(0.2151)
Academic performance ranking		0.03214
		(0.1292)
Political affiliation		−0.02069
		(0.08719)
Average monthly household income		−0.03919
		(0.09441)
Rounds		−0.03856
		(0.02817)
Constant term	0.1001	−0.2432
	(0.1056)	(0.4891)
Sample size	700	700
Adj. *R*^2^ (Pseudo *R*^2^)	0.0042	0.0736

** *p < 0.05 and*

*** *p < 0.01*.

In addition, Table 4 compares the differences in the accuracy of information possessed by individuals between treatments. Because it does not pass the normality test (Treatment 1: *z* = 8.277, *p* < 0.01; Treatment 2: *z* = 8.621, *p* < 0.01) and the homogeneity test of variance (Chi-square = 10.1566, *p* < 0.01), it is subjected to the Wilcoxon rank-sum test. The results show that comparing with no feedback, when individuals’ information sharing behavior can get positive information feedback, their information search behavior show low significant change, so there is nearly no difference in their information accuracy.

#### Reciprocity Norms and Information Sharing Behavior

In order to verify whether the influence of feedback on individual information sharing behavior works by reciprocity norms, we first explore the relationship between reciprocity norms and information sharing behavior. Reciprocity norms are measured using the average of participants’ three reciprocity norm questions. Then, the consistency of participants’ reciprocity norms between different treatments is analyzed. Results show that there is low significant differences in participants’ reciprocity norms between the two treatments (treatment 1 vs. treatment 2: 4.5741 vs. 4.4118, *t* = 1.4160, *p* = 0.1572, diff 95% CI = [−0.0627, 0.3874]). In order to verify the influence of reciprocity norms on information sharing behavior, the participants’ reciprocity norm score and information sharing behavior are, respectively, taken as the independent and dependent variables for Logit regression. The regression results are shown in regression 1 of Table 6. Furthermore, the results of sub-sample regression for treatments 1 and 2 are shown in regressions 2 and 3.

**Table 6:** Reciprocity norms and information sharing behavior.

Dependent variable: information sharing behavior			
Independent variable	Full sample	Treatment 1	Treatment 2	Full sample
	Regression 1	Regression 2	Regression 3	Regression 4
Reciprocity norms	0.4805^***^	0.1611^*^	1.0269^***^	0.1656^**^
	(0.06147)	(0.08382)	(0.1123)	(0.08204)
Treatment				−2.8030^***^
				(0.6444)
Reciprocity norms×Treatment				0.7662^***^
				(0.1371)
Gender	0.6700^***^	1.4383^***^	0.6013^*^	0.7786^***^
	(0.1940)	(0.3930)	(0.3196)	(0.2081)
Grade	0.5773^***^	1.5128^***^	−0.2310	0.6387^***^
	(0.1965)	(0.3481)	(0.3973)	(0.2242)
Major	−0.6100^***^	−0.4889	−0.01664	−0.3218
	(0.1919)	(0.3561)	(0.2933)	(0.2107)
Work experience	0.1354	−0.1621	−0.04141	0.1209
	(0.2161)	(0.4940)	(0.3519)	(0.2281)
Academic performance ranking	0.2480^*^	−0.6810^***^	0.7541^***^	0.1752
	(0.1348)	(0.2247)	(0.2406)	(0.1353)
Political affiliation	−0.1549	−0.003278	−0.2274	−0.2115^**^
	(0.09423)	(0.1599)	(0.1689)	(0.09722)
Average monthly household income	−0.09894	−0.02665	−0.006698	−0.1463
	(0.09820)	(0.1575)	(0.1602)	(0.09922)
Constant term	−2.7269^***^	−1.8909^**^	−5.0961^***^	−1.4386^**^
	(0.5506)	(0.9328)	(0.8942)	(0.6480)
Sample size	700	360	340	700
Adj. *R*^2^	0.1316	0.1617	0.2880	0.1821

(1) Information sharing behavior is determined by whether the participant is willing to share the searched information to the information receivers in the same group in the phase 2 (unwilling to share = 0; willing to share = 1). Treatment is a dummy variable of experiment treatment (treatment 1 = 0; treatment 2 = 1). (2) ^*^p < 0.1, ^**^p < 0.05, and ^***^p < 0.01.

Regression 1 in Table 6 shows that individuals with higher reciprocity norms are more inclined to share information. The width of the 95% confidence interval which is [0.3600, 0.6009] is small enough to confirm the effect of the association. Regression 3 show that when getting feedback, individuals’ reciprocity norms have a significant positive effect on the individuals’ information sharing behavior. However, we can see that from the Regressions 2, when taking the reciprocity norms as the independent variable and information sharing behavior as the dependent variable, the 95% confidence interval which is [−0.0032, 0.3254] indicates that the association is weak. That is, the role of reciprocity norms may vary in different treatments.

To further verify the difference in the effect of the reciprocity norms between treatments, we perform regression on the interaction term between reciprocity norms and treatments. The results are shown in regression 4 in Table 6. It can be seen that compared with no feedback, reciprocity norms have a stronger role in promoting individual information sharing behavior when information sharer receives feedback. The width of the 95% confidence interval of the interaction term which is [0.4974, 1.0350] is small to confirm the effect of the association. On the whole, the improvement effect of feedback on individual information sharing behavior is realized by strengthening the reciprocity norms.

#### Interaction Between Information Accuracy and Reciprocity Norms

The previous analysis concludes that the more accurate the information an individual has, the more willing he/she is to share information. This part attempts to analyze whether this conclusion is affected by the reciprocity norms. Next, the information sharing behavior is used as the dependent variable. We perform Logit regression on the interaction term between the information accuracy and reciprocity norms. The results are shown in regression 1 in Table 7. Additionally, sub-sample regression is performed for treatments 1 and 2, respectively, and the results are shown in regressions 2 and 3.

**Table 7:** Information accuracy, reciprocity norms, and information sharing behavior.

Dependent variable: information sharing behavior		
Independent variable	Full sample	Treatment 1	Treatment 2
	Regression 1	Regression 2	Regression 3
Reciprocity norms	0.2424^**^	−0.04897	0.5967^***^
	(0.1057)	(0.1445)	(0.1791)
Information accuracy	−0.09058^*^	−0.006729	−0.3581^***^
	(0.05178)	(0.06388)	(0.1157)
Reciprocity norms×Information accuracy	0.02028^**^	0.008323	0.06321^***^
	(0.009678)	(0.01171)	(0.01992)
Gender	0.7681^***^	1.6635^***^	0.9652^***^
	(0.1958)	(0.4279)	(0.3558)
Grade	0.6709^***^	1.3486^***^	0.2703
	(0.2049)	(0.3479)	(0.4569)
Major	−0.4955^**^	−0.5425	0.1206
	(0.1981)	(0.3450)	(0.3134)
Work experience	0.09829	0.1184	0.09003
	(0.2184)	(0.4863)	(0.3428)
Academic performance ranking	0.2245^*^	−0.7423^***^	0.6655^***^
	(0.1341)	(0.2224)	(0.2464)
Political affiliation	−0.1883^**^	−0.09729	−0.3839^**^
	(0.09353)	(0.1587)	(0.1839)
Average monthly household income	−0.09397	0.04193	−0.06875
	(0.1009)	(0.1664)	(0.1527)
Constant term	−1.8757^***^	−0.9959	−2.8941^***^
	(0.6495)	(1.1642)	(1.0751)
Sample size	700	360	340
Adj. *R*^2^	0.1421	0.1801	0.3117

* *p < 0.1,*

** *p < 0.05, and*

*** *p < 0.01*.

Regression 1 show that, in general, the higher the reciprocity norms of individuals, the higher the accuracy of the information they have, and the more willing they are to share information (coef. = 0.02028, *p* = 0.036, 95% CI = [0.0013, 0.0393]). Combining regression 2 with the previous analysis, we can know that the interaction between reciprocity norms and information accuracy disappears when there is no feedback and slightly weaker reciprocity norms. Regression 3 indicates that individuals with higher reciprocity norms are more willing to share accurate information when receiving feedback (coef. = 0.0632, *p* < 0.01, 95% CI = [0.0242, 0.1022]).

## Discussion

Information accuracy has become one of the core issues worthy of attention in the field of information sharing. Especially in the current situation of rapid innovation of Internet technology and highly developed social environment, the information accuracy is directly related to the security and stability of society. Therefore, it is crucial to study individuals’ preferences for sharing accurate information and the possible underlying mechanisms. To shed light on this issue, we conduct a set of laboratory experiments to study people’s willingness to share accurate information. To be specific, an experiment is designed with information accuracy endogenous, that is, an information search step is added to enable people to control the accuracy of the information they search. Meanwhile, for investigating the role of reciprocity norms in willingness to share accurate information, the reciprocity degree of participants is measured in our experiments. Additionally, we set a treatment with feedback, in which the role of the reciprocity norms is enhanced by the feedback from the information receivers. The experimental results can further illustrate the role of reciprocity norms on accurate information sharing.

The first contribution of our manuscript is to provide evidence supporting the view that when people obtain more accurate information, they are more willing to share it (Nicolaou et al., 2013; Pennycook et al., 2020). Furthermore, in our experiments, people are able to control the accuracy of the information they have. Overall speaking, our results support the view that people share inaccurate information not because people do not have preferences for sharing accurate information (Van Bavel and Pereira, 2018), nor because people have difficulty judging the accuracy of information (Serra-Garcia and Gneezy, 2021), but rather a failure to let information accuracy guide sharing decisions (Altay et al., 2022). This view logically confirms the effectiveness of the currently advocated nudge strategy to suppress misinformation sharing by guiding people to consider the accuracy of the information they obtain (Pennycook et al., 2021; Roozenbeek et al., 2021).

We further examine the promotion effect of reciprocity norms on people’s accurate information sharing behavior. First, the results suggest that individuals with higher reciprocity norms are more likely to share information, which is consistent with previous research (Haeussler, 2011; Schumann et al., 2014; Pai and Tsai, 2016). Moreover, the promotion effect stems from people’s expectation of returns (Kim et al., 2021), that is, people expect to get positive return in the future because of information sharing behavior. The more accurate the information people have, the higher the perceived usefulness of the information (Larcker and Lessig, 1980; Machdar, 2019). Sharing more useful and accurate information, people expect higher reciprocal benefits in the future. Thus, individuals with higher reciprocity norms are more willing to share accurate information which is consistent with our findings.

Furthermore, our results show that the effect of reciprocity is not obvious in treatment 1 without feedback, but significant when the information receiver can give positive feedback on the obtained information. The possible reason is that positive feedback makes participants perceive themselves to be evaluated by others, thus reinforces the role of reciprocity (Lee and Suzuki, 2020), finally promotes their behavior to share accurate information (Li and Sakamoto, 2015). In this way, the role of reputation as a bridge between reciprocity and sharing accurate information is highlighted. In treatment 2, getting feedback is a direct reputation mechanism which promotes individuals’ information sharing behavior. Participants with a higher reciprocity give higher weight to reputation feedback based on reciprocity norms when sharing information, and thus prefer to share accurate information.

Our study examines the explanation power of the reciprocity norms in an accurate information share area which can provide a basis for future research. This study helps to develop methods to promote the information accuracy in the information sharing, i.e., enhancing individuals’ reciprocity norms compliance and strengthening the effect pathway of the reciprocity norms.

## Conclusion

We conduct an “information search—information sharing” and an “information search—information sharing —information feedback experiment” to examine individuals’ behavior of sharing accurate information and its influencing factors. Our results indicate that individuals who are willing to obtain accurate information prefer to share information. We also find that information feedback can enhance accurate information sharing preference through reciprocity norms. That is, individuals with higher reciprocity norms are more willing to share accurate information. This study captures individuals’ behavior and preference characteristics with regard to the accuracy of the information they share in the era of highly developed network interaction.

## Limitations and Future Directions

This research has several limitations. First, the number of information searches is used as a proxy for the accuracy of the information people have, which makes the information accuracy mixed with the individuals’ efforts. Individuals are more willing to share information that they have put more efforts into, which is a potential explanation. In future studies, the possible roles of efforts and information accuracy should be separated. Second, our research focuses on the validation of the unilateral result that people are more willing to share accurate information. In the future, we will additionally examine whether people are willing to share inaccurate information that they get. On the basis of this study, we will increase the types of information to examine the relationship between the accuracy of other information and the sharing behavior of individuals except the information related to people’s vital interests. Third, as all the regressions, the parametric tests, and the nonparametric test contribute to one conclusion of our manuscript. Thus, the null hypotheses of these tests are tested in parallel which requires the use of *p*-value adjustments theoretically. However, the conclusion of this manuscript tends to be a qualitative judgment. Thus no formal adjustment of all *p*-values is used in our manuscript (Greenland and Hofman, 2019). The absence of such an adjustment is a possible limitation of our manuscript. Finally, this study does not examine what mechanisms nudge people’s willingness to share accurate information. For example, recent research has shown that directing people’s attention to the accuracy of information is an effective boosting mechanism for accurate information sharing. Further, the potential of gamification for motivating people to share their information has been recognized by a growing amount of studies conducted in recent years. Gamification can serve as a nudge, in the sense of applying choice architecture to push people to select desired behavior works well, to improve the individuals’ information sharing or even accurate information sharing behaviors. In the following research, we can analyze and verify these mechanisms, and seek other effective mechanisms.

## Data Availability Statement

The raw data supporting the conclusions of this article will be made available by the authors, without undue reservation.

## Ethics Statement

The studies involving human participants were reviewed and approved by Ethics Committee of the Shandong University of Finance and Economics. The patients/participants provided their written informed consent to participate in this study.

## Author Contributions

KL conceived the idea of the manuscript and designed the research. KL and WX collected and analyzed the data and wrote the manuscript. All authors contributed to the article and approved the submitted version.

## Funding

This study was supported by the Natural Science Fund of Shandong Province (grant no: ZR2021QG055).

## Conflict of Interest

The authors declare that this research was conducted in the absence of any commercial or financial relationships that could be construed as a potential conflict of interest.

## Publisher’s Note

All claims expressed in this article are solely those of the authors and do not necessarily represent those of their affiliated organizations, or those of the publisher, the editors and the reviewers. Any product that may be evaluated in this article, or claim that may be made by its manufacturer, is not guaranteed or endorsed by the publisher.
